# Cognitive flexibility: neurobehavioral correlates of changing one’s mind

**DOI:** 10.1093/cercor/bhac431

**Published:** 2022-11-11

**Authors:** Katharina Zühlsdorff, Jeffrey W Dalley, Trevor W Robbins, Sharon Morein-Zamir

**Affiliations:** Department of Psychology, University of Cambridge, Downing Place, Cambridge, CB2 3EB, United Kingdom; The Alan Turing Institute, British Library, 96 Euston Road, London, NW1 2DB, United Kingdom; Behavioural and Clinical Neuroscience Institute, Department of Psychology, University of Cambridge, Downing Street, Cambridge, CB2 3EB, United Kingdom; Department of Psychology, University of Cambridge, Downing Place, Cambridge, CB2 3EB, United Kingdom; Behavioural and Clinical Neuroscience Institute, Department of Psychology, University of Cambridge, Downing Street, Cambridge, CB2 3EB, United Kingdom; Department of Psychiatry, University of Cambridge, Herchel Smith Building, Forvie Site, Robinson Way, Cambridge, CB2 0SZ, United Kingdom; Department of Psychology, University of Cambridge, Downing Place, Cambridge, CB2 3EB, United Kingdom; Behavioural and Clinical Neuroscience Institute, Department of Psychology, University of Cambridge, Downing Street, Cambridge, CB2 3EB, United Kingdom; School of Psychology and Sport Science, Anglia Ruskin University, East Road, Cambridge, CB1 1PT, United Kingdom

**Keywords:** anterior insula, anterior cingulate cortex, flexibility, proactive control, uncertainty

## Abstract

Behavioral and cognitive flexibility allow adaptation to a changing environment. Most tasks used to investigate flexibility require switching reactively in response to deterministic task-response rules. In daily life, flexibility often involves a volitional decision to change behavior. This can be instigated by environmental signals, but these are frequently unreliable. We report results from a novel “change your mind” task, which assesses volitional switching under uncertainty without the need for rule-based learning. Participants completed a two-alternative choice task, and following spurious feedback, were presented with the same stimulus again. Subjects had the opportunity to repeat or change their response. Forty healthy participants completed the task while undergoing a functional magnetic resonance imaging scan. Participants predominantly repeated their choice but changed more when their first response was incorrect or when the feedback was negative. Greater activations for changing were found in the inferior frontal junction, anterior insula (AI), anterior cingulate, and dorsolateral prefrontal cortex. Changing responses were also accompanied by reduced connectivity from the AI and orbitofrontal cortices to the occipital cortex. Using multivariate pattern analysis of brain activity, we predicted with 77% reliability whether participants would change their mind. These findings extend our understanding of cognitive flexibility in daily life by assessing volitional decision-making.

## Introduction

Cognitive flexibility is defined as the ability to adapt to changes in the environment by switching task sets, responses, or strategies (Cools 2015). From a psychological perspective, it is often considered alongside related emergent control processes such as inhibition and updating (Miyake et al. 2000). Flexibility allows changing behavior in response to environmental and internal cues. Individuals with greater cognitive flexibility have improved life outcomes, better social functioning, and reduced cognitive decline with age (Koesten et al. 2009; Diamond and Lee 2011; Burke et al. 2019). Difficulties in flexibility have also been observed in several mental health conditions, further highlighting the importance of research on the neural and psychological bases of flexible behavior (Remijnse et al. 2006; Chamberlain 2007; Yerys et al. 2009).

A full understanding of cognitive flexibility has proven challenging, in part because this is not a unitary construct (Dias et al. 1996). Various behavioral paradigms have been developed to measure flexibility in humans, each capturing intersecting but somewhat different aspects of this behavior (Milner 1963; Roberts et al. 1988; Dajani and Uddin 2015). Common paradigms encompass task-switching, attentional set-shifting, and reversal learning (Brown et al. 2015). In task-switching procedures, the participant switches between two or more tasks based on predefined stimulus–response sets (Monsell 2003; Manoach 2009). Set-shifting tasks, such as the Wisconsin Card Sorting Test (WCST), the CANTAB intradimensional/extradimensional test, or the Dimensional Change Card Sort task, measure the ability to transition between cognitive-attentional sets in the case of the WCST by using external cues to learn about rules determining ongoing performance (Grant and Berg 1948; Chamberlain 2007). Reversal learning, on the other hand, requires participants to adapt responding following the reversal of previously learnt reward-related contingencies (Izquierdo et al. 2017). The diversity within cognitive flexibility is exemplified by distinct developmental trajectories for different tasks (Somsen 2007; Kalkut et al. 2009; Van Der Schaaf et al. 2011; Buttelmann and Karbach 2017).

In studies involving both task-switching and set-shifting paradigms, the salience and executive control networks (ECN) are consistently activated (Dajani and Uddin 2015; Uddin 2021). Key nodes in the former include the anterior insula (AI) and dorsal anterior cingulate cortex (dACC), whereas the ECN includes the inferior frontal junction (IFJ), dorsolateral prefrontal cortex (dlPFC), ventrolateral prefrontal cortex (vlPFC), and inferior frontal gyrus (IFG) (Hampshire and Owen 2006; Braun et al. 2015; Shine et al. 2016; Vaghi et al. 2017). Connectivity between the AI, dACC, and thalamus, forming the cingulo-opercular network, in addition to the posterior parietal cortex is widely reported in task-switching (Dosenbach et al. 2006; Liston et al. 2006; Worringer et al. 2019). While the neural substrates of task-switching and attentional set-shifting overlap, the former is associated with greater parietal and thalamic involvement and the latter is associated with the default mode network (DMN) (Vatansever et al. 2016). Reversal learning, on the other hand, activates somewhat different circuits, including the striatum, amygdala, the medial PFC, and the lateral and medial orbitofrontal cortex (OFC) (Remijnse et al. 2005; Izquierdo et al. 2017). Feedback learning and establishing outcome probabilities are important components of task performance on numerous flexibility paradigms. In human functional magnetic resonance imaging (fMRI) studies, the dlPFC and subcortical structures, such as the dorsomedial striatum, signal uncertainty and expectancy deviations that are necessary for learning and updating probabilities in a given context (Lopez-Paniagua and Seger 2013; Mestres-Missé et al. 2017). This is further supported by data from nonhuman primates showing active dlPFC neural populations during the initial stages of a reversal learning task, differing distinctly from signals after a reversal (Bartolo and Averbeck 2020).

Cognitive control more generally has been increasingly recognized to employ proactive and reactive modes (Braver 2012). Applying this distinction to cognitive flexibility may be useful with task-switching paradigms often focusing on reactive flexibility and switching being triggered by explicit external signals. Proactive control, on the other hand, anticipates the need for control and is thought to be mediated in part by the lateral PFC (lPFC) (Braver et al. 2009; Mäki-Marttunen et al. 2019). Both reactive and proactive control involve vlPFC and AI node activations, though only proactive control engages the DMN (Ryman et al. 2019). A psychophysiological interaction (PPI) analysis, which allows the investigation of connectivity from a preselected region of interest (ROI) during an event, has linked proactive control to greater intra-ECN connectivity compared to greater within salience network (SN) connectivity in reactive control (Wang et al. 2015). By contrast, reversal learning and attentional set-shifting paradigms characteristically capture both reactive and proactive flexibility, with shifting being both prepared and elicited by feedback in participants (Rogers et al. 2000). There are additional key procedural differences between the common instantiations of these paradigms. For example, many task-switching procedures include similar proportions of switch and repeat trials, whereas most reversal learning and behavioral set-shifting procedures employ fewer shifts (see also Dodds et al. 2011). Less frequent task relevant events elicit the SN (Wager et al. 2004). Additional procedural distinctions include the extent of rule learning during the task and presentation of feedback or rewards to signal a need to change, both of which are less prominent in task-switching paradigms (Kiesel et al. 2010).

In daily life, flexibility often involves a change in behavior being generated endogenously but in situations where rule learning or set-shifting is not required. Here, we report results from a “change your mind” task, where participants performed two-alternative forced choices and following spurious feedback, viewed, and responded to the same stimulus again. Critically, they could repeat their previous response or change it, acting of their own volition. To our knowledge, no existing task provides participants with the opportunity to repeat their choice and to assess whether they subsequently choose the “road not taken.” We propose that the ability to change one’s responses constitutes a form of mental flexibility not fully explored within current conceptual frameworks (Uddin 2021). We assessed “changing one’s mind” performance, its neural correlates, including neural connectivity and possible predictors during the task. Forty healthy participants completed the task while undergoing fMRI scanning. We expected subjects to change their second response when their first response was incorrect or when feedback was negative and that any effects of spurious feedback would decrease with time. Based on previous studies, it was hypothesized that PFC salience and ECN nodes (i.e. AI, dACC, IFJ, IFG, and dlPFC) would be active when participants volitionally “changed their minds.” PPI analyses investigated connectivity differences from 4 different ROIs during change versus repeat trials. We expected stronger connectivity between the striatum and vlPFC on change trials, reflecting response inhibition. Multivariate pattern analyses (MVPAs) use pattern classification algorithms to categorize neuroimaging data based on spatial and/or temporal patterns and have been used to predict behavior on a trial-by-trial basis (Li et al. 2014; Huang et al. 2021). We hypothesized that the MVPA classifier would be able to predict a change with high accuracy based on feedback and the first response.

## Methods

### Participants

Participants were 40 healthy volunteers (21 females, handedness [right–left]: 37–3), aged between 18 and 60 years (mean = 31.88, standard deviation [SD] = 10.03), recruited from adverts in the Cambridge community and from the University of Cambridge Behavioural and Clinical Neuroscience Institute volunteer panel. None of the participants had any history of psychiatric or neurological disorders. Verbal IQ was assessed using the National Adult Reading Test score (mean = 115.29, SD = 8.68) (Nelson 1982). All participants provided informed written consent and were reimbursed for their time and travel expenses, with ethical approval granted from the Cambridge Local Research Ethics Committee (08/H0308/65).

### Task procedure

**Fig. 1:**
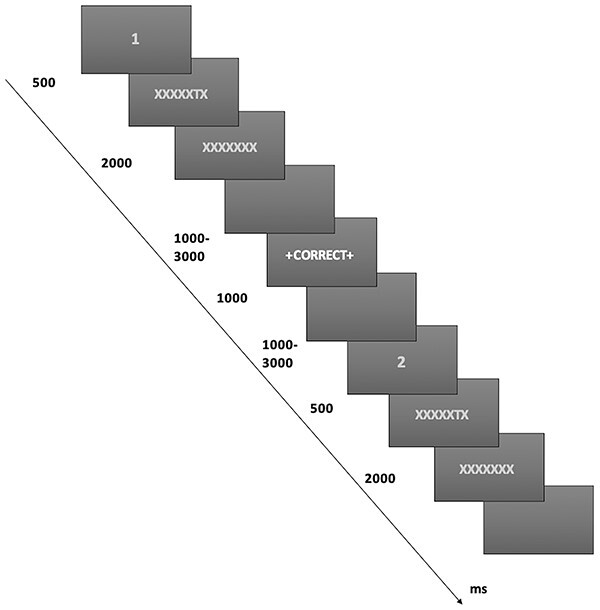
Diagrammatic representation of the task structure. In pairs of trials, a target letter (“T” or “L”) was presented and masked with an “X” after a predetermined duration. The total stimulus duration of 2,000 ms was comprised of a brief target presentation and a subsequent mask. After responding, participants received feedback that was orthogonal to their performance. This was followed by an identical second stimulus presentation. The first and second of each pair were denoted by fixation appearing as the numeral “1” or “2.” event durations are shown to the left of the task representation.

Participants performed a two-alternative forced choice task, determining whether a target letter was “T” or “L” (see Fig. 1). The task consisted of paired trials, each of which included an identical stimulus display following a fixation (“1” or “2”) denoting whether this was the first or second of the pair. The target letter was embedded in a row of Xs and masked after a brief delay. Participants were informed that, after the first of each pair, the computer will provide feedback but that the feedback may not always be 100% accurate. Instructions stated that, immediately after the feedback, the same trial will be presented again and that they would have a chance to change their mind on the second display should they wish. Unbeknown to participants, feedback was orthogonal to performance and was monitored so that it was negative on half of accurate trials and positive on half of the incorrect trials.

Participants completed 56 pairs of trials in each of three runs, 48 of which were difficult and 8 were easy. On each trial, following a 500-ms fixation period, the target was briefly presented within a row of 8 Xs in gray in the center of the screen. Following a predetermined brief duration, the target was replaced with an “X.” Target location was counterbalanced, appearing in 1 of the 6 or 4 central locations on difficult or easy trials, respectively. On difficult trials, a staircase determined target duration, initially set to 60 ms and decreasing by 10 ms following a correct response and similarly increasing following an incorrect response. On easy trials, target duration was fixed at 150 ms. The stimuli displayed for a total of 2 s, and participants responded by pressing 1 of 2 buttons on a custom button-box with response mappings counterbalanced across participants. Following 1–3 s (duration randomly selected at 100-ms intervals), feedback appeared for 1 s (“+ correct +” in green or “x wrong x” in red). After another interval (1–3 s), the second fixation appeared, followed by the same stimulus display and an intertrial interval (1–2.5 s). This procedure allowed for 6.2 s on average between the 2 responses of each pair. Participants completed 12 practice pairs before entering the scanner. Trial order per block was randomized and the experiment was programmed in Visual Basic.

### Neuroimaging acquisition

Participants were scanned on a 3 Tesla Siemens MAGNETOM Trio scanner at the Wolfson Brain Imaging Centre (Cambridge, UK). After the acquisition of a T1-weighted (T1w) scan, Siemens standard echoplanar imaging sequence was used depicting blood oxygenation level-dependent (BOLD) contrast, with time repetition = 2,000 ms, flip angle = 78°, time echo = 30 ms, in an interleaved ascending sequence. The field of view was 192 × 192 mm, with matrix 64 × 64, echo spacing = 0.47 ms, and bandwidth = 2,442 Hz/Px. Volume number per run varied from 285 to 336. Each image volume comprised 32 slices of 3-mm thickness, with in-plane resolution of 3 × 3 mm, orientated parallel to the anterior commissure–posterior commissure line.

### Task performance

The main measure of interest was mean change on response 2 (R2), with accuracy and reaction time (RT) as secondary measures. Linear mixed-effects (LME) models were fit with feedback (positive or negative), response 1 accuracy (R1: correct or incorrect), run (1–3), and all interactive terms as factors in R (R Core Team, 2017, version 4.0.4) using the lme4 package (Bates et al. 2015). Subsequent post hoc pairwise comparisons of estimated marginal means with Tukey adjustment were conducted using the emmeans package (Lenth 2018; Pinheiro et al. 2021). Various LMEs were tested that included different random intercepts and were subsequently compared using the Akaike information criterion, Bayesian information criterion, and log likelihood. The LME, including a random intercept for each subject and run, had the lowest scores across all 3 measures and is thus reported here. The winning model was: mean change R2 ~ feedback × R1 accuracy × run + (1 + run|subject).

### Imaging data

#### Preprocessing

Preprocessing of fMRI data was performed using FMRIB Software Library (FSL) (Smith et al. 2004) and FMRIPREP, a Nipype-based tool (Esteban et al. 2018). Each T1w volume was corrected for intensity nonuniformity using N4BiasFieldCorrection and skull-stripped using antsBrainExtraction with the OASIS template in the Advanced Normalisation Tools software (Tustison et al. 2010). Spatial normalization to the ICBM 152 Nonlinear Asymmetrical template version 2009c was performed through nonlinear registration with the antsRegistration tool using brain-extracted versions of both T1w volume and template (Avants et al. 2008). Brain tissue segmentation of cerebrospinal fluid, white matter, and gray matter was performed on the brain-extracted T1w using fast (FSL) (Zhang et al. 2001). fMRI data were slice-time-corrected using slicetimer (FSL) and motion-corrected using mcflirt (FSL) (Jenkinson et al. 2002). Distortion correction was performed using field maps processed with fugue (FSL) (Jenkinson 2003). This was followed by coregistration to the corresponding T1w image using boundary-based registration with 6° of freedom using flirt (FSL) (Greve and Fischl 2009). The field distortion correcting warp, BOLD-to-T1w transformation and T1w-to-template (Montreal Neurological Institute [MNI]) warp were concatenated and applied in a single step using antsApplyTransforms using Lanczos interpolation.

Frame-wise displacement was calculated for each functional run using the implementation of Nipype (Power et al. 2014). The first 5 volumes were discarded to avoid T1 saturation effects. The images were subsequently high-pass filtered (128 s) and spatially smoothed with a 6-mm full-width, half-maximum 3D Gaussian kernel. A canonical hemodynamic response function was modeled to the onsets of the explanatory event types. Quality checks were conducted by checking successful registration, ensuring that none of the participants showed excessive motion using DVARS and framewise-displacement measures (excessive motion threshold being 10% of the total number of volumes) and by inspecting their respective carpet plots. All subjects passed these quality checks.

#### First-level model

A first-level fMRI linear model was fit using FEAT (FSL) (Woolrich et al. 2001) for each run and included the following event types: (i) R1 correct, (ii) R1 incorrect, (iii) positive feedback, (iv) negative feedback, (v) R2 change, and (vi) R2 repeat. Equivalent event types for easy trials and 6 movement parameters (*x*, *y*, *z*, pitch, roll, and yaw) resulting from the image realignment to control for movement artifacts were also included. Contrasts included only difficult trials and consisted of: (i) R1 incorrect versus correct, (ii) negative versus positive feedback, and (iii) R2 change versus repeat.

#### Higher level models

A primary second-level model averaged the first-level models for each subject across the 3 runs. An additional model included the effect of run to assess changes over time on the 6 event types included in the first-level model. The contrasts above were subsequently examined in third-level mixed-effects whole-brain analyses involving one-sample *t*-tests with cluster thresholding with a *Z*-threshold of 3.1 and *P* < 0.05 to correct for multiple comparisons (Woolrich et al. 2004). Figures of fMRI results were created using FSLeyes (Smith et al. 2004).

#### Connectivity analysis

To further investigate the circuits involved in changing or repeating a behavior, a whole-brain PPI analysis was conducted in FSL. Four ROIs were created based on the findings from the first analyses as well as from areas reported to mediate proactive cognitive flexibility in previous studies. The regions that were selected for PPI analysis included the caudate nucleus, AI, OFC, and superior frontal gyrus (SFG). A 5-mm sphere was placed in the center of the respective region based on the Harvard-Oxford Atlas. Mean timeseries for each participant and each ROI were extracted. AI and SFG were selected due to their involvement in error monitoring and because they are part of the SN and ECN, respectively, which are both active in task-switching and set-shifting paradigms (Magno et al. 2006; Uddin 2021). The OFC, on the other hand, is involved in learning of response-outcome contingencies and reversal learning tasks (Schoenbaum et al. 2007). Finally, the caudate nucleus was included due to its role in response inhibition and feedback learning (Tricomi and Fiez 2008; Schmidt et al. 2020).

A PPI analysis tests whether there is an interaction between the ROI timeseries and a cognitive process which accounts for the neural responses observed in other brain regions (Neufang et al. 2008). This was tested by including the extracted time series, the response to the cognitive process of interest (change vs. repeat), and an interaction term between them. Contrasts tested the differences in connectivity in the change versus the repeat condition. One-sample *t*-tests for each seed-region were conducted and clusters above a *Z*-threshold of 2.5 and *P* < 0.05 were considered to be significant.

**Fig. 2:**
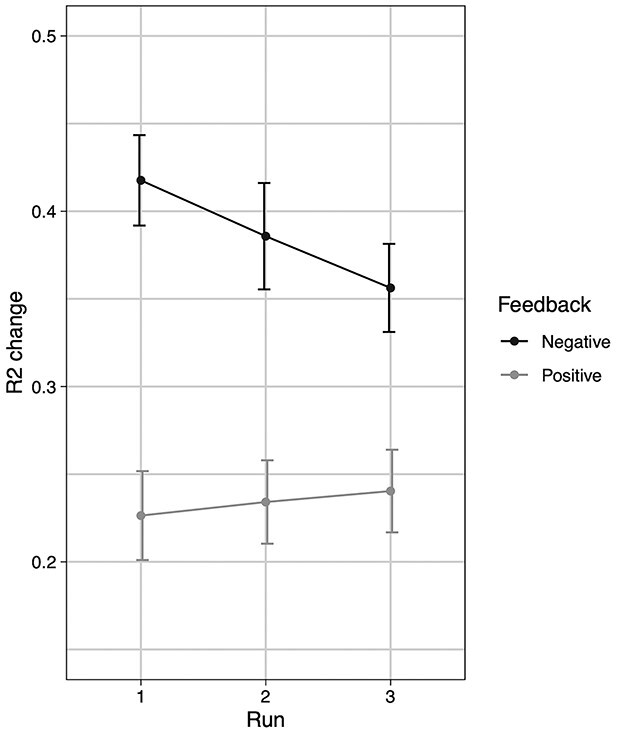
Effect of feedback and run on R2 change. The LME model for R2 change had significant feedback and feedback × run effects across both accurate and inaccurate R1 responses. Participants changed more following spurious negative feedback compared with after spurious positive feedback, though this effect was reduced across the 3 runs. Error bars represent standard error of the mean.

**Fig. 3:**
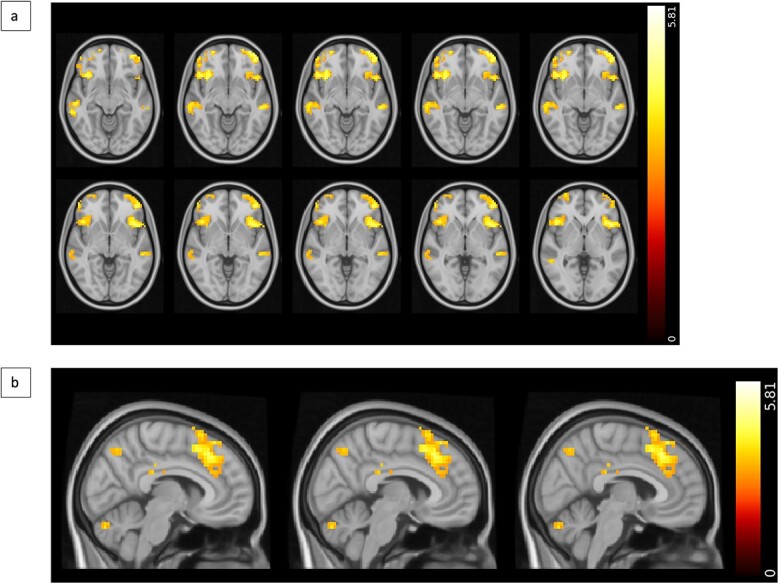
Results summary of change versus repeat contrast. a) Axial slices (MNI *z* = −10 to −1) of the contrast highlighting key regions activated more when participants changed compared to when they repeated their responses. b) Sagittal view (MNI *x* = 6–4) of the contrast highlighting key regions activated more when participants changed compared to when they repeated their responses. Activations detected with a whole-brain analysis involving one-sample *t*-tests with cluster thresholding with a *Z*-threshold of 3.1 and *P* < 0.05. The bar on the right represents the *t*-statistic.

#### Multivariate pattern analysis

An MVPA employing a support vector machine (SVM) algorithm was used to predict whether participants would change their minds or repeat their response during R2. The MVPA was run on nonnormalized and unsmoothed data to reduce distortions and was conducted using nilearn and sklearn in Python 3.6 (Pedregosa et al. 2011; Abraham et al. 2014). The MVPA was based on the whole-brain individual beta maps obtained from the univariate analysis, more specifically, the beta maps for feedback, R1, and beta maps for the 2 categories were combined by concatenation. The individual beta maps were represented as separate points in a high-dimensional space in which a linear decision boundary was determined through a hyperplane. The decision boundary separated the scans based on the labels assigned to them, in our case, changing or repeating a response on the next trial. The classifier identified a hyperplane that could best separate the provided input space (Li et al. 2014). The SVM applied a linear kernel, rather than a nonlinear kernel to minimize overfitting, using a fixed regularization parameter (*C* = 1). This parameter *C* controls the trade-off between having no training errors and allowing misclassifications and was set to 1 based on previous publications (Yang et al. 2019; Vilgis et al. 2022). Cross-validation was performed using the leave-one-out method. This method excludes 1 subject from each group, trains the classifier on the remaining data, and then tests performance on the excluded participants. This procedure is repeated for each subject. Subsequently, the classification process was repeated 1,000 times with a different random permutation of the training labels. The purpose of permutation testing was to determine whether the overall classification accuracy was statistically significant and was used to derive a *P*-value. Performance was evaluated using (i) the F1 score, (ii) the area under the receiver operating characteristic curve (AUROC), and (iii) classification accuracy. Maps visualizing decoder weights showed which areas contributed to classifying whether the subject would change or repeat their choice on the subsequent stimulus presentation.

## Results

### R2 change performance

Analysis of mean R2 change revealed that it was significantly affected by feedback (*F*(1, 394) = 31.15, *P* < 0.001, η^2^*P* = 0.07) and R1 accuracy (*F*(1, 394) = 16.93, *P* < 0.001, η^2^*P* = 0.04). Mean R2 change was higher following negative feedback (*M* = 0.39, SD = 0.24) compared with after positive feedback (*M* = 0.23, SD = 0.22). Mean R2 change was also higher when R1 was incorrect (*M* = 0.41, SD = 0.24) compared with when R1 was correct (*M* = 0.21, SD = 0.19). The interaction between R1 accuracy and feedback was not significant (*F*(1, 394) = 2.18, *P* = 0.14, η^2^*P* = 0.0055). Additionally, a feedback × run interaction was evident (*F*(1, 394) = 3.96, *P* = 0.047, η^2^*p* = 0.01), as seen in Fig. 2. Participants increasingly disregarded negative feedback with time (*t*(394) = −2.00, *P* = 0.047 for negative feedback but not for positive feedback (*t*(394) = −0.40, *P* = 0.69). The 3-way interaction between R1 accuracy, feedback, and run was not significant (*F*(1,394) = 1.33, *P* = 0.25, η^2^*p* = 0.0034).

### Brain activations when changing versus repeating a response

Figure 3 shows that the R2 change-repeat contrast revealed significantly greater activity in the paracingulate/cingulate gyrus, anterior insular cortex, superior, middle, and IFGs, superior and middle temporal gyri, OFC, frontal pole, and caudate as well as in the lateral occipital cortex and cerebellum when a participant changed their response (Table 1). Both repeating and changing a response were associated with significant activations in the SFG, paracingulate gyrus, cingulate gyrus, insular cortex, OFC, caudate, putamen, thalamus, frontal pole, intracalcarine and supracalcarine cortices, and cerebellum.

**Table 1:** Summary of fMRI peak activity for the contrast of R2 change versus repeat. Results from a whole-one-sample *t*-tests with cluster thresholding with a *Z*-threshold of 3.1 and *P* < 0.05.

Name	BA	Side	MNI coordinates (*X*, *Y*, *Z*)	Number of voxels	Volume (mm^3^)	Mean *z*-statistic
Frontal pole	10	R	30, 64, −4	367	12,386	3.49
Frontal pole	10	L	−35, 59, −5	199	6,716	3.60
Anterior insular cortex	13,16	R	38, 20, 2	96	3,240	3.76
Anterior insular cortex	13,16	L	−40, 20, 2	86	2,903	3.75
OFC	11,47	R	42, 26, −7	167	5,636	3.67
OFC	11,47	L	−50, 25, −8	119	4,016	3.73
SFG	6,8	R	3, 36, 44	251	8,471	3.64
SFG	6,8	L	−7, 27, 47	168	5,670	3.85
Middle frontal gyrus	46	R	47, 35, 31	252	8,505	3.53
Middle frontal gyrus	46	L	−48, 18, 42	93	3,139	3.39
Paracingulate gyrus	32	R	3, 29, 42	168	5,680	3.71
Paracingulate gyrus	32	L	−7, 40, 27	168	5,680	3.80
Anterior cingulate gyrus	24	R	8, 30, 24	104	3,510	3.58
Anterior cingulate gyrus	24	L	−6, 28, 27	67	2,261	3.46
IFG	44,45	R	45, 20, 9	182	6,142	3.50
IFG	44,45	L	−48, 18, 5	161	5,434	3.55
Middle temporal gyrus	21	R	58, −25, −6	92	3,105	3.50
Middle temporal gyrus	21	L	−59, −25, −6	34	1,147	3.58
Angular gyrus	39	R	51, −53, 43	294	9,922	3.72
Angular gyrus	39	L	−55, −54, 43	363	12,251	3.95
Supramarginal gyrus	40	R	56, −44, 45	225	7,594	3.58
Supramarginal gyrus	40	L	−57, −46, 45	295	13,331	3.89
Lateral occipital cortex	19	R	38, −63, 45	163	5,501	3.88
Lateral occipital cortex	19	L	−37, −61, 45	275	9,281	3.96
Precuneus cortex	7	R	6, −68, 45	11	371	3.38
Precuneus cortex	7	L	−6, −73, 45	38	1,282	3.50

Note. BA - Brodmann area.

### Neural connectivity when repeating versus changing a response

The PPI analyses highlighted interactions between brain areas involved in modulating responses in the presented paradigm. The change versus repeat contrast of the AI PPI analysis showed there was greater connectivity between the AI and occipital areas when repeating compared to changing R2 (Table 2). These areas include the lateral occipital cortex and occipital pole lateralized to the left hemisphere. Similarly, there was stronger connectivity in the repeat condition between the OFC and occipital areas, such as the lingual gyrus, occipital pole, and occipital fusiform gyrus. Enhanced connectivity between the SFG and the left posterior cingulate gyrus and precuneus cortex was found when changing compared to repeating R2 (see Supplementary Materials). There were no differences in the change versus repeat contrast when the PPI analysis with the caudate nucleus seed.

**Table 2:** Summary of fMRI peak activity for the PPI contrast of change versus repeat with AI as the seed-region. Results from a whole-brain analysis involving one-sample *t*-tests with cluster thresholding with a Z-threshold of 2.5 and *P* < 0.05.

Name	BA	Side	MNI coordinates (X, Y, Z)	Number of voxels	Volume (mm^3^)	Mean *z*-statistic
Lateral occipital cortex	19	L	−30, −82, 24	58	1,958	2.87
Occipital pole	18	L	−30, −95, 19	32	1,080	2.85

Note. BA = Brodmann area.

**Table 3:** Summary of performance metrics for the MVPA analysis. Performance of 4 analyses is compared. The first column represents classification accuracy purely based on behavior. In the second, classification was based purely on the beta maps from the first response. The third analysis used the beta maps from the feedback conditions, and the last analysis combined the 2 by concatenating the beta maps. Performance was measured using the accuracy, AUROC, and F1 scores.

		Model		
Measure	Performance	Response 1	Feedback	Response 1 + feedback
Accuracy	0.70	0.64	0.74	0.77
AUROC	0.64	0.57	0.83	0.86
F1	0.68	0.66	0.74	0.78

### MVPA: predicting changing one’s mind

Using an MVPA approach, we tested whether it is possible to predict whether participants would change or repeat their response. R2 change could be predicted from brain activity during R1, presentation of feedback, as well their combination. Analyses using R1 beta coefficient maps yielded an accuracy of 0.64 predicting R2 change, and those using the feedback resulted in an accuracy of 0.74 (*P* = 0.013 and *P* < 0.001, respectively). When R1 and feedback were combined, performance was superior with an accuracy of 0.77 (*P* < 0.001). For comparability, LME model accuracy for behavioral performance was 0.70. Table 3 presents a summary of performance metrics, including the AUROC and F1 scores. The maps visualizing decoder weights gave an indication of regions contributing to classifying whether participants would repeat or change their response (Supplementary Fig. S1). Based on R1 activity, voxels contributing to R2 classification encompassed the cingulate, caudate, putamen, inferior and middle frontal gyri, frontal pole, and occipital cortex, with additional voxels distributed throughout the brain. When the feedback beta maps were used, areas involved in classification included the AI and OFC, as well as the SFG, paracingulate gyrus, cingulate gyrus, caudate, and frontal pole.

### Additional performance measures

Mean R1 accuracy was 0.59 (SD = 0.071, 95% CI [0.56, 0.61]), indicating participants found the task challenging to complete. Mean R1 accuracy was significantly affected by run as subjects’ performance improved (*F*(1, 79) = 4.35, *P* = 0.040). Mean R2 accuracy was higher at 0.64 (SD = 0.071, 95% CI [0.61, 0.66]). An analysis of R2 accuracy indicated significant R1 accuracy and R1 accuracy × feedback effects (*F*(1, 394) = 403.2, *P* < 0.001, η^2^*P* = 0.51 and *F*(1, 394) = 63.28, *P* < 0.001, η^2^*P* = 0.14, respectively). Mean R2 accuracy was significantly higher following correct R1 compared with incorrect R1 (*M* = 0.78, SD = 0.19; *M* = 0.40, SD = 0.25, respectively). When R1 was correct, R2 accuracy was higher when feedback was positive (*t*(394) = −5.06, *P* < 0.001). However, when R1 was incorrect, R2 accuracy was lower when feedback was positive (*t*(394) = 6.19, *P* < 0.001). There were no effects of run on mean R2 accuracy (*F*(1, 472) = 0.90, *P* = 0.34).

Additionally, an analysis of mean RT2 revealed a decrease across runs (*F*(1, 39) = 9.88, *P* = 0.0032), and an interaction between feedback and R1 accuracy (*F*(1, 394) = 4.21, *P* = 0.041), with faster responding when R1 was correct and feedback was positive compared to when R1 was incorrect and feedback was negative (*t*(394) = 5.23, *P* < 0.001; *M* = 632.2 ms, SD = 178.8 ms; *M* = 686.1 ms, SD = 197.7, respectively). When comparing RT2s for correct trials followed by negative feedback with incorrect trials followed by positive feedback, no differences were found (*t*(235) = 0.91, *P* = 0.36).

### Activations associated with R1 response and with feedback

Greater activity when R1 was incorrect versus correct was found in the paracingulate, cingulate, and superior frontal gyri and the right angular gyrus (Supplementary Table S1). Brain activations associated with R1, when R1 was either correct or incorrect, were observed in the cingulate and paracingulate gyri, superior, middle, and inferior frontal gyri, insular cortex, lateral occipital cortex, and superior parietal lobule.

When assessing activations associated with feedback, the BOLD response was greater for negative than positive feedback presentation in the superior frontal and superior temporal gyri, with no other differences between the two conditions (Supplementary Table S2). Activations associated with feedback, either positive or negative, were observed in the superior and middle temporal gyri, occipital cortex, and right frontal pole.

### Changes over time

**Fig. 4:**
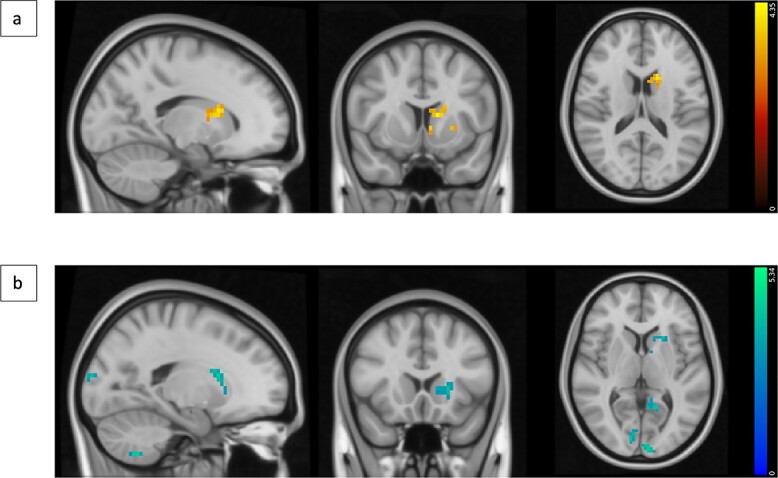
Change in signal in response to feedback with run. a) Voxels that positively correlated with run, i.e. show an increased signal in response to positive feedback with each run. The voxels are in the left striatum (MNI coordinates: *X* = −13, *Y* = 9, *Z* = 13). b) Voxels positively correlated with run when negative feedback is presented. The voxels were found in the left striatum, occipital pole, and cerebellum (MNI coordinates: *X* = −19, *Y* = 17, *Z* = 3). Results were obtained via a mixed-effects whole-brain analysis involving one-sample *t*-tests with cluster thresholding with a *Z*-threshold of 3.1 and *P* < 0.05.

Exploratory analyses assessed whether activations associated with any of the 6 events of interest changed over the 3 runs. In both the R2 change and repeat conditions, activity in the occipital pole increased with run. Over time, activity to both positive and negative feedback in the left dorsal striatum increased (Fig. 4). With negative feedback, an increase in the frontal pole, occipital pole, and cerebellum was also seen (Table 4). With correct R1, frontal pole and ventromedial PFC activations increased across runs. When R1 was incorrect, the dorsal striatum signal bilaterally decreased with time.

**Table 4:** Summary of fMRI peak activity following positive and negative feedback across runs. Results from a whole-brain analysis involving one-sample *t*-tests with cluster thresholding with a *Z*-threshold of 3.1 and *P* < 0.05.

Name	BA	Side	MNI coordinates (*X*, *Y*, *Z*)	Number of voxels	Volume (mm^3^)	Mean z-statistic
Positive feedback						
Caudate		L	−13, 10, 14	39	1,316	3.55
Putamen		L	−24, 10, −1	15	506	3.43
Negative feedback						
Caudate		L	−15, 12, 14	50	1,687	3.63
Putamen		L	−23, 12, −2	38	1,282	3.73
Occipital pole	18	R	15, −92, 14	51	1,721	3.63
Occipital pole	18	L	−10, −98, 11	90	3,037	3.65
Lingual gyrus	19	R	11, −82, 2	17	573	3.51
Lingual gyrus	19	L	−10, −50, 2	39	1,316	3.57
Precuneus cortex	7	R	9, −62, 16	19	641	3.53
Precuneus cortex	7	L	−1, −71, 25	28	945	3.50
Cerebellum		L	−21, −61, −50	26	877	3.37
Frontal pole	10	R	3, 54, −2	12	405	3.27
Frontal pole	10	L	−4, 58, −2	20	675	3.50

Note. BA = Brodmann area.

## Discussion

This study investigated behavioral and neural correlates of proactive cognitive flexibility, where participants adjusted their behavior voluntarily. Specifically, we assessed flexibility under conditions that did not involve trial-and-error rule-based learning or switching between multiple task-rules or attentional sets. Instead, participants were allowed a “do-over,” illuminating a perspective on flexibility not fully captured to date. Using paired trials in an uncertain environment, participants decided whether to change or repeat their response in the second of each pair. Accuracy of performance for the first of each pair was only slightly above chance, indicating that uncertainty was high. As expected, participants were more likely to change their response following the presentation of the second stimulus when the first response was incorrect and when the feedback was negative.

The focus of the findings was on changing one’s mind on the second response and its neural correlates. We noted ACC activation associated with R2 change in addition to R1 responses. Activation during the first response, particularly when incorrect, is consistent with previous studies which identified this region along with the SFG as key structures involved in error and action monitoring (Carter et al. 1998; Botvinick et al. 1999; Van Veen et al. 2001; Lie et al. 2006; Bonini et al. 2014). Electrophysiological evidence from primates has shown that ACC neurons not only respond to errors but also signal prior to adjustments following an error, thereby contributing to the selection of the next movement (Shima and Tanji 1998). Further, ACC neuronal activity reflects a behavioral shift on the subsequent trial in a reversal learning task (Kawai et al. 2015). Lesioning or inactivating this area impairs the ability to adjust choice behavior after negative outcomes in both experimental animals and humans (Williams et al. 2004). Studies in humans have focused on neural correlates of error signaling or detection (Van Veen and Carter 2002; Ullsperger et al. 2014). The present findings complement these by focusing on the subsequent behavioral adjustment, where strong ACC and SFG activations were detected, i.e. on the instantiation of flexibility through a change on the second response. This indicates the importance of the ACC during the “implementation” of behavioral adjustments, which has hitherto received less attention in humans (Ridderinkhof et al. 2004). The ACC and SFG involvements also demonstrate the inherent conceptual overlap between error monitoring and flexibility in some everyday situations, with error monitoring preceding flexibility.

In line with other studies of behavioral shifting, we report the recruitment of PFC regions of the ECN and SN (e.g. IFJ, IFG, AI, ACC, and dlPFC) during response change (Braun et al. 2015; Shine et al. 2016; Dajani et al. 2020). The IFJ is thought to mediate response shifting by integrating multiple behavioral control processes and interactions with the dlPFC, vlPFC, AI, and putamen, additional evidence for which is provided by our findings (Cole and Schneider 2007; Levy and Wagner 2011; Sundermann and Pfleiderer 2012). The AI and IFG, on the other hand, are both important for response inhibition and contribute in dissociable ways (Cai et al. 2014). The former is functionally coupled with the ACC and is important for salience detection of unexpected or infrequent events. Involvement of the AI is unlikely to be due to the detection of an unexpected stimulus as in the case of inhibition in stop signal or go/no-go tasks. Instead, given that changing was more infrequent than staying, it may be associated with the salience of an internally generated infrequent action. The IFG has stronger connectivity with the dlPFC as part of the ECN and facilitates appropriate behavioral control (Cai et al. 2014). The recruitment of both the AI and IFG may be attributable to inhibition of the first response, highlighting the intrinsic role of inhibition in purposeful flexible adjustment of behavior. Unlike previous studies investigating flexibility, we also observed activity in the frontal pole in both change and repeat conditions, with greater activations during the former. This may represent a volitional component of our task, as this area has been associated with processing of internal states and self-generated responses (Christoff et al. 2003; Ramnani and Owen 2004; Tsujimoto et al. 2010).

Our PPI analysis provided additional insight into the brain circuits underlying repeating or changing a response. With the AI as the seed region, there was stronger connectivity to the lateral occipital cortex and occipital pole in the former condition. This was also the case for the connectivity from the OFC to the occipital pole, lingual gyrus, and occipital fusiform gyrus. Further, occipital pole activation increased over time during the second response. A recent automated meta-analysis of cognitive shifting, updating, and inhibition highlighted not only the importance of prefrontal regions but also the angular gyrus and visual cortex, whose role in cognitive flexibility is often overlooked (Uddin 2021). Reports from multiple task-switching studies further confirm the involvement of occipital regions such as the lateral occipital cortex (Armbruster et al. 2012; Dajani et al. 2020). In mice, ACC projection neurons to the visual cortex important for error monitoring have been directly linked to posterror behavioral adaptation through fiber photometry and optogenetic manipulations (Norman et al. 2021). In humans, a causal dependency between the frontal operculum and occipitotemporal regions in cognitive control has also been demonstrated using transcranial magnetic stimulation, suggesting top-down regulation of visual areas by the AI and frontal operculum (Higo et al. 2011). Whether occipital areas in our task were solely activated due to the stimuli being visual is unclear. Nevertheless, our findings support an involvement of the AI/OFC and connections to occipital regions in flexible cognitive control.

MVPA is increasingly used to increase the information gained from fMRI data (Weaverdyck et al. 2020). In the present study, using the combined activity during the first response and the feedback presentation, the algorithm could predict the participants’ next action with an accuracy of 77%. As the prediction of behavior from performance data in this tightly controlled situation was 70%, the BOLD voxel-wise approach enhanced the predictive properties of the data. When predictions were based solely on activations during feedback presentation, accuracy was 74%, compared to 64% when beta maps from the first response were used. This suggests that both internal monitoring and external feedback contribute to the change of mind, with feedback appearing to have greater weighting here, likely due to the high level of uncertainty introduced by the brief presentation time. There was insufficient evidence for the interaction of the two even though they would be expected to in daily life. Future research should aim to investigate such interactions further. Decoder weight maps highlighted voxels in the AI, OFC, frontal pole, SFG, cingulate gyrus, and caudate as strongly contributing to predicting a change response on the subsequent stimulus presentation. These results indicate that brain activity prior to an action reflects the antecedents of changing one’s mind.

Over time, participants changed their responses less following negative feedback, suggesting they learnt it was unreliable. This was accompanied by increased activity over time in the dorsal striatum during feedback presentation, regardless of valence, in addition to the increased activity in the occipital cortex during the second response mentioned above. These findings are compatible with the established role of the dorsal striatum in behavioral and cognitive control, decision-making under uncertainty, and signaling ambiguity (Hsu et al. 2005; Lopez-Paniagua and Seger 2013; Mestres-Missé et al. 2017). Thus, the dorsal striatum has been found to estimate outcomes and adjust behavior when there are deviations from expectations (Mestres-Missé et al. 2014). Specifically, the dorsal striatum and its connections with cortical regions, such as the ECN, are necessary for overriding previous responses that are no longer useful, and instead, for implementing more appropriate responses given the current environmental context (Den Ouden et al. 2010; Mestres-Missé et al. 2014, 2017). Caudate nucleus activation has been shown to correlate with response selection rather than feedback presentation (Hiebert et al. 2017). Here, the increased dorsal striatal activity during feedback presentation over time likely reflects its involvement in using ambiguous information to update response selection, a finding that has not been reported in previous studies.

This study created high uncertainty via brief stimulus presentation times and spurious feedback. Though effective in eliciting participants to change their minds, the task could not dissociate specific contributions of internal versus external sources of uncertainty, with the former reflecting an individual’s internal state and the latter reflecting feedback from the environment. Future studies may disentangle these or explore their interactions along with alternative contributors to shifting present in everyday situations. The task addresses difficulties encountered by the task impurity problem, which arise from executive functions necessarily operating on other cognitive processes and make it difficult to untangle joint and separable aspects of executive processes (Miyake et al. 2000; Uddin 2021). Thus, one challenge in studying cognitive flexibility has been its isolation from working memory and attention demands. These processes were likely kept constant in the change versus repeat contrast, with the task having minimal spatial and rule learning requirements. Future studies could employ the same task with auditory stimuli to verify modality independent involvement of key PFC nodes. Moreover, this could reveal whether occipital region activation is due to the visual nature of most flexibility tasks (Niendam et al. 2012). Ultimately, the present findings broaden our view of flexibility in everyday life by enabling a targeted assessment of volitional decision-making.

## Supplementary Material

Supplementary_materials_bhac431
