# Supplementary material for: Cognitive flexibility: neurobehavioral correlates of changing one’s mind
Source: Cereb Cortex. 2022 Nov 11;33(9):5436–46. doi: 10.1093/cercor/bhac431 (PMC10152092; doi:10.1093/cercor/bhac431)
Supplement: Supplementary_materials_bhac431 [file supplementary_materials_bhac431.docx]

**Supplementary Materials**

| **Name** | **BA** | **Side** | **MNI coordinates (X, Y, Z)** | **Number of voxels** | **Volume (mm^3^)** | **Mean z-statistic** |
| --- | --- | --- | --- | --- | --- | --- |
| Paracingulate Gyrus | 32 | R | 3, 35, 33 | 19 | 641 | 3.36 |
| Paracingulate Gyrus | 32 | L | -3, 29, 38 | 38 | 1283 | 3.64 |
| Superior Frontal Gyrus | 6,8 | R | 3, 22, 50 | 17 | 574 | 3.37 |
| Superior Frontal Gyrus | 6,8 | L | -3, 27, 50 | 36 | 1215 | 3.64 |
| Angular Gyrus | 39 | R | 60, -52, 27 | 25 | 844 | 3.48 |
| Anterior Cingulate Gyrus | 24 | R | 3, 30, 34 | 11 | 371 | 3.45 |
| Anterior Cingulate Gyrus | 24 | L | -2, 20, 38 | 11 | 371 | 3.66 |

**Supplementary Table 1.** Results summary of incorrect vs correct R1 contrast.

Table of all areas that were found two differ between incorrect and correct R1 including their respective statistics and anatomical information. BA – Brodmann area; MNI – Montreal Neurological Institute template.

| **Name** | **BA** | **Side** | **MNI coordinates (X, Y, Z)** | **Number of voxels** | **Volume (mm^3^)** | **Mean z-statistic** |
| --- | --- | --- | --- | --- | --- | --- |
| Superior Frontal Gyrus | 6,8 | R | 7, 18, 64 | 28 | 945 | 3.63 |
| Superior Frontal Gyrus | 6,8 | L | -1, 19, 60 | 3 | 101 | 3.40 |
| Superior Temporal Gyrus | 41, 42 | R | 50, -24, -5 | 38 | 1282 | 3.46 |

**Supplementary Table 2.** Results summary of negative vs positive feedback contrast.

Table of all areas that were found two differ between positive and negative feedback including their respective statistics and anatomical information. BA – Brodmann area; MNI – Montreal Neurological Institute template.

| **Name** | **BA** | **Side** | **MNI coordinates (X, Y, Z)** | **Number of voxels** | **Volume (mm^3^)** | **Mean z-statistic** |
| --- | --- | --- | --- | --- | --- | --- |
| Lingual Gyrus | 17,18 | L | -8,-86,-5 | 47 | 1586 | 2.84 |
| Occipital Pole | 18 | R | -9, 93, -4 | 18 | 608 | 2.82 |
| Occipital Pole | 18 | L | -5, -93, -5 | 45 | 1519 | 2.89 |
| Occipital Fusiform Gyrus | 37 | L | -19, -88, -11 | 33 | 1114 | 2.84 |

**Supplementary Table 3.** Summary of fMRI peak activity for the PPI contrast of change vs repeat with OFC as the seed-region.

Results from a whole-brain analysis involving one-sample t-tests with cluster thresholding with a Z-threshold of 2.5 and p<0.05.

| **Name** | **BA** | **Side** | **MNI coordinates (X, Y, Z)** | **Number of voxels** | **Volume (mm^3^)** | **Mean z-statistic** |
| --- | --- | --- | --- | --- | --- | --- |
| Cingulate Gyrus, posterior | 23, 31 | L | -4, -47, 26 | 42 | 1418 | 2.81 |
| Precuneus Cortex | 7 | R | 4, 61, 38 | 8 | 280 | 2.66 |
| Precuneus Cortex | 7 | L | -3, -61, 28 | 44 | 1485 | 2.82 |

**Supplementary Table 4.** Summary of fMRI peak activity for the PPI contrast of change vs repeat with SFG as the seed-region.

Results from a whole-brain analysis involving one-sample t-tests with cluster thresholding with a Z-threshold of 2.5 and p<0.05.


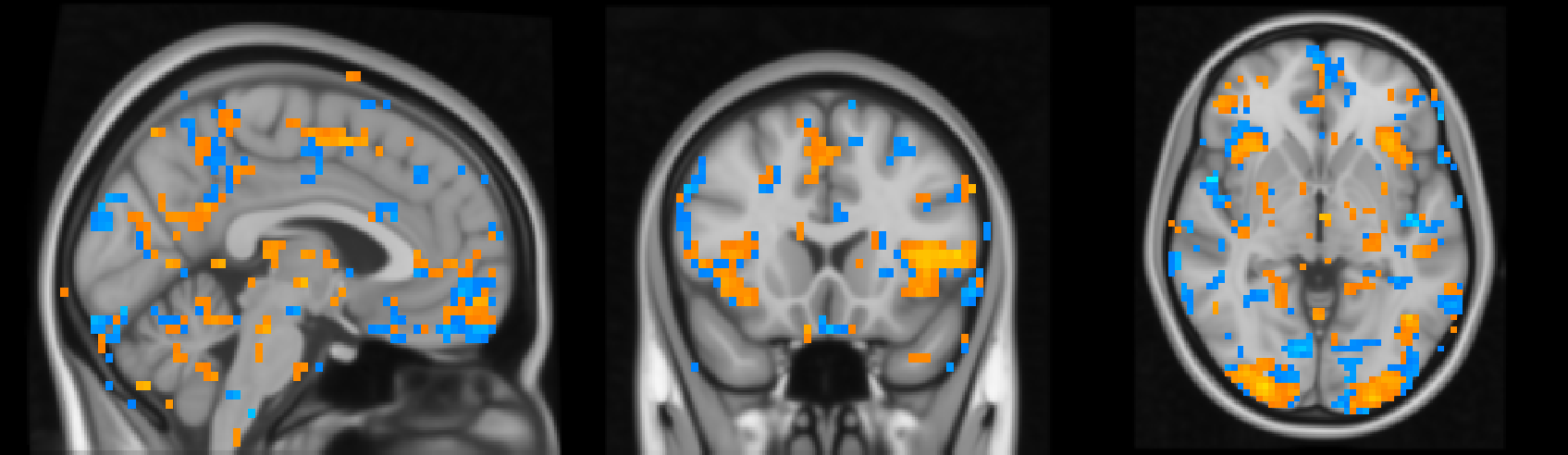
**Supplementary Figure 1. Multivariate Pattern Analysis results.** MVPA decoder weights for change and repeat conditions based on beta maps during the feedback presentation. The voxels shown contributed to the decoder deciding whether the participant would change or repeat their response on the next stimulus presentation. Blue voxels aided in the classification of a repeat trial, orange voxels helped classify a change trial. MNI coordinates: X=-5, Y=15, Z=4.
